# Molecular Characterization of Xyloglucanase *cel74a* from *Trichoderma reesei*

**DOI:** 10.3390/ijms22094545

**Published:** 2021-04-27

**Authors:** Douglas Christian Borges Lopes, Cláudia Batista Carraro, Roberto Nascimento Silva, Renato Graciano de Paula

**Affiliations:** 1 Molecular Biotechnology Laboratory, Department of Biochemistry and Immunology, Ribeirao Preto Medical School (FMRP), University of Sao Paulo, Ribeirao Preto 14049-900, SP, Brazil; douglas.chr.94@gmail.com (D.C.B.L.); claudiacarraro@usp.br (C.B.C.); renato.paula@ufes.br (R.G.d.P.); 2 Department of Physiological Sciences, Health Sciences Centre, Federal University of Espirito Santo, Vitoria 29047-105, ES, Brazil

**Keywords:** *Trichoderma reesei*, xyloglucanase, sugarcane bagasse (SCB), holocellulase, xyloglucan breakdown

## Abstract

Background: The filamentous fungus *Trichoderma reesei* is used on an industrial scale to produce enzymes of biotechnological interest. This fungus has a complex cellulolytic system involved in the degradation of lignocellulosic biomass. However, several aspects related to the regulation of the expression of holocellulolytic genes and the production of cellulases by this fungus are still understood. Methods: Here, we constructed a null mutant strain for the xyloglucanase *cel74a* gene and performed the characterization of the Δ*cel74a* strain to evaluate the genetic regulation of the holocellulases during sugarcane bagasse (SCB) cultivation. Results: Our results demonstrate that the deletion of xyloglucanase *cel74a* may impact the regulation of holocellulase expression during SCB cultivation. The expression of cellulases *cel7a*, *cel7b*, and *cel6a* was reduced in Δ*cel74a* strain, while the hemicellulases *xyn1* and *xyn2* were increased in the presence of SCB. The *cel74a* mutation also affected the xyloglucan hydrolysis patterns. In addition, CEL74A activity was modulated in the presence of calcium, suggesting that this ion may be required for efficient degradation of xyloglucan. Conclusions: CEL74A affects the regulation of holocellulolytic genes and the efficient degradation of SCB in *T. reesei*. This data makes a significant contribution to our understanding of the carbon utilization of fungal strains as a whole.

## 1. Introduction

Lignocellulosic biomass is one of the most abundant renewable sources of polysaccharides on the planet with a particular abundance in agroindustrial wastes [1,2,3]. This is of particular interest because of its potential application in the production of biofuels and other bioproducts. Lignocellulosic biomass is formed particularly with high amount in both cellulose and hemicellulose, which means that all processes hoping to use this waste product as a carbon source need to include enzymes that can degrade these polysaccharides [4,5]. While cellulose is a homopolymer consisting only of glucose molecules linked by β-(1→4) bonds, hemicellulose is a group of heteropolysaccharides made up of different monosaccharides bound by different types of bonds [6,7]. Xyloglucan is one of the main hemicelluloses found in the cell wall of both dicots and non-gramineous monocots and is associated with cellulose via hydrogen bonds [8]. Hemicellulose is composed of a β-1,4-D-glucose backbone and α-(1,6)-linked xylosyl residue branches which may include β-(1,2)-linked galactosyl, arabinosyl, and fucosyl residues [9,10,11,12].

Several microorganisms have been shown to catalyze the bioconversion of lignocellulosic biomass via regulated gene expression and the production of a wide range of hydrolytic and oxidative enzymes [13,14,15,16]. Holocellulase expression in *Trichoderma reesei* is regulated in a carbon source-dependent manner and induced by various activators including cellobiose, lactose, sophorose, xylobiose, and xylose, which trigger the production of holocellulolytic enzymes that degrade lignocellulosic biomass [17]. One of these Carbohydrate Active Enzymes (CAZymes—http://www.cazy.org/, accessed on 24 April 2021) is xyloglucanase (CEL74A—Xyloglucanase from *Trichoderma reesei*), which is responsible for the cleavage of the β-(1→4) bonds between the glucose molecules in the xyloglucan backbone [18,19,20,21]. It was also previously reported that xyloglucanases from different microorganisms can cleave xyloglucan in different positions and act on certain motifs in xyloglucan backbone releasing different products [9,22,23,24]. Xyloglucan acts as a protective barrier of cellulose against osmotic stress and enzymatic action. So, its degradation by CEL74A allows the exposure of this cellulosic fraction and plays an important role in the lignocellulosic biomass degradation by exposing and accelerating the enzymatic cellulose fraction degradation by cellulases [25,26,27].

In *T. reesei*, secretome analysis after induction on sugarcane bagasse, cane molasses medium (CMM), and lactose-based conventional medium (LCM) showed that xyloglucanase CEL74A is one of the most abundant enzymes (a member of Glycosyl hydrolase family 74—GH74 family) in the supernatant culture of *T. reesei* [25,28,29]. Additionally, Dos Santos Castro et al. [21] showed, for the first time, that xyloglucanase CEL74A is enriched in *T. reesei* secretome in the presence of cellulose. Interestingly, Dos Santos Castro et al. [21,30] demonstrated that xyloglucanase *cel74a* gene is upregulated when *T. reesei* QM9414 is grown in the presence of cellulose and sophorose. In addition, De Paula et al. [31] identified that xyloglucanase CEL74A is at least sevenfold induced in *T. reesei* QM6a grown in the presence of sugarcane bagasse when compared to glycerol as sole carbon source. Together, these results suggest that CEL74A plays an essential and still unknown role in lignocellulosic degradation in filamentous fungi. Thus, we selected the gene encoding to xyloglucanase CEL74A for characterization using functional genomic and others molecular studies.

In this context, we constructed a mutant strain by deletion of the main xyloglucanase CEL74A of *T. reesei* producing Δ*cel74a*. This strain was then characterized in terms of its phenotypic changes, its effect on holocellulase transcription and enzyme activity during the growth in the presence of sugarcane bagasse (SCB). Finally, we tried to understand the indirect influence of this xyloglucanase on the regulation of holocellulase gene expression in this fungus.

## 2. Results

### 2.1. Phylogenetic Analysis and 3D Structure Analysis

**Figure 1:**
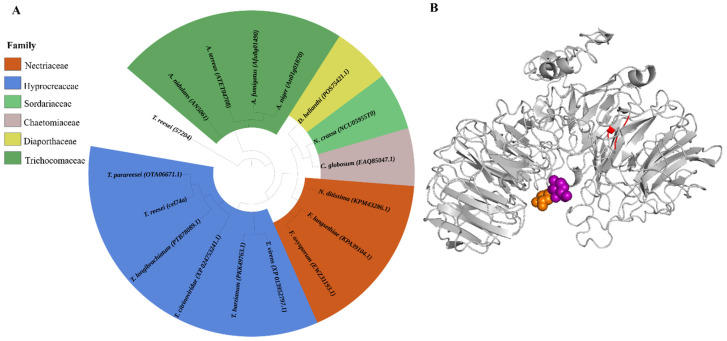
Phylogenetic relationships between the orthologous GH74 protein sequences and the xyloglucanase CEL74A protein from *T. reesei* and its predicted 3D structure. (**A**) Maximum-parsimony phylogenetic tree of conserved xyloglucanase protein sequences. These alignments were performed using the MUSCLE algorithm. The radial tree was generated using the maximum-likelihood method and built by the iTOL program [32,33]. (**B**) Predicted three-dimensional structures of xyloglucanase CEL74A with the highlighted sections representing specific interaction domains: the calcium-binding domain (red), glucose-binding domain (purple), and xylose-binding (orange), respectively.

The protein encoded by *Tr49081* is described as a glycoside hydrolase family 74 (GH 74—xyloglucanase *cel74a*) protein in the annotated genome of *T. reesei,* available from the JGI Genome Portal (https://mycocosm.jgi.doe.gov/Trire2/Trire2.home.html, accessed on 24 April 2021). Phylogenetic analysis of *Tr49081* (xyloglucanase—CEL74A) and other fungal glycosyl hydrolases indicated that *Tr49081* demonstrated a high degree of homology to other GH74 sequences including the hydrolases of *Trichoderma longibrachiatum, T. harzianum, T. citronoviridae, T. virens*, and *T. parareesei*, as well as other GH74 proteins from various unrelated fungi such as *Fusarium oxysporum*, *F. langsethiae*, *Neurospora crassa*, *Diaporthe helianthin*, *Neonectria ditissima*, *Chaetomium globosum*, *Aspergillus terreus*,* A. niger*, *A. fumigatus*, and *A. nidulans* (Figure 1A).

The 3-D structural analysis demonstrated that xyloglucanase CEL74A from *T. reesei* has a predicted calcium-binding conserved domain (highlighted in red) and is comprised of a set of β-sheets surrounded by loops and helices (Figure 1B). The model produced a C-score of −1.65, a TM-score of 0.51 ± 0.15 and an RMSD (Root Mean Square deviation) of 12.6 ± 4.3 Å for *N. crassa*; a C-score of −0.57, a TM-score of 0.64 ± 0.13, and an RMSD of 9.8 ± 4.6 Å for *C. thermophilus*; a C-score of 0.23, a TM-score of 0.74 ± 0.11, and an RMSD of 7.9 ± 4.4 Å for *Aspergillus nidulans*; and a C-score of 0.86, a TM-score of 0.61 ± 0.14, and an RMSD of 10.5 ± 4.6 Å for *Trichoderma reesei*, respectively.

### 2.2. Xyloglucanase CEL74A Activity is Regulated by Calcium

**Figure 2:**
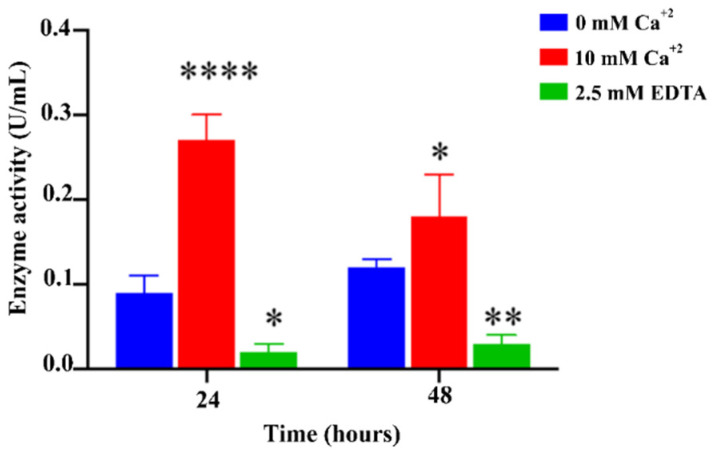
Xyloglucanase activity in the supernatants after 24 and 48 h of cultivation in the presence of SCB, 10 mM CaCl_2_, and 2.5 mM EDTA (Ethylenediamine tetraacetic acid). **** (*p* < 0.0001), ** (*p* < 0.01) and * (*p* < 0.05).

Our 3D structural prediction of *T. reesei* CEL74A revealed that this enzyme has a conserved calcium-binding domain (Figure 1B), suggesting that its enzyme activity may be regulated by Ca^2+^. In order to assess the importance of calcium in CEL74A activity, we performed an enzyme assay using 10 mM calcium and 2.5 mM EDTA as a chelating agent. Figure 2 clearly shows that the xyloglucanase activity in the parental strain increased in the presence of calcium and is significantly impaired in the presence of EDTA at both 24 h and 48 h. This result suggests that functional CEL74A possesses a calcium binding site that regulates *T. reesei* xyloglucanase activity.

### 2.3. cel74a Gene Is Upregulated in T. reesei in the Presence of SCB

**Figure 3:**
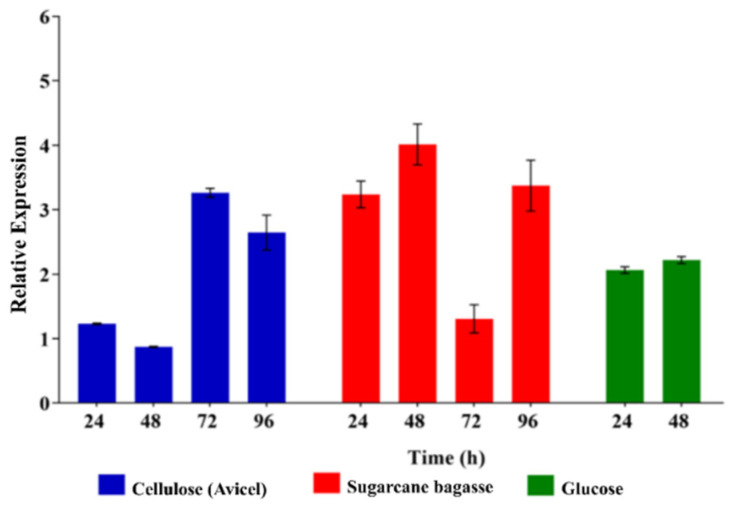
Expression profile of xyloglucanase *cel74a* in the QM6a parental strain grown in cellulose, SCB, and glucose. Expression levels were calibrated using the comparative 2^−ΔCt^ method (for glucose), and 2^−ΔΔCt^ (for cellulose and SCB) using the constitutively expressed *Sar1* GTPase gene as an endogenous control, and glycerol as a control group, respectively. These results are based on three replicates from three independent experiments and are expressed as mean ± standard deviation.

Previous results showed that the xyloglucanase *cel74a* gene is upregulated when *T. reesei* QM9414 is grown in the presence of cellulose, sophorose, or sugarcane bagasse [21,30,31]. Here, we analyzed xyloglucanase *cel74a* gene expression in the QM6a parental strain grown in the presence of SCB, cellulose or glucose a known repressor of cellulase synthesis. As demonstrated in Figure 3, the xyloglucanase *cel74a* gene was expressed at low levels in the presence of cellulose (Avicel), with a marked increase at 72 and 96 h of cultivation. Gene expression in the presence of SCB was evaluated at 24, 48, 72, and 96 h of cultivation (Figure 3), and the highest expression levels were observed at 24, 48, and 96 h. No significant change in gene expression was observed over time in the presence of glucose (Figure 3). Moreover, the expression levels of *cel74a* at shorter induction times on glucose are higher than the control on glycerol (another repressive carbon source), reinforcing the idea that glucose not repressing transcription of this gene. Together, this result suggests that xyloglucanase *cel74a* is not regulated via catabolite repression in *T. reesei*. Furthermore, the increased expression of *cel74a* observed in the longest times of cellulose induction might be related to the signaling mechanisms involved in the regulation of the gene expression of *cel74a* in response to the cellulose fraction. However, further experiments are necessary to investigate the additional regulatory mechanisms controlling this process.

The *T. reesei* Δ*cel74a* strain was constructed using homologous recombination in the QM6aΔ*tmus53*Δ*pyr4* parental strain, whose non-homologous end joining pathway was disrupted [34]. The correct integration of the deletion cassette in this strain was verified by PCR (Table S1 and Figure S2A) and in this case, integration at the homologous locus resulted in a specific amplicon with a size of approximately 1.8 kb and 1.6 kb (Figure S2B) depending on the exact location of the outside primer. Additionally, no amplification was observed in the mutant strain when a pair of primers that annealed inside the coding region of *cel74a* were used. An amplicon of 1.078 kb was detected only in the parental strain (Figure S2C) and no *cel74a* mRNA was detected in the mutant strain (Figure S2D). These results confirmed the complete deletion of the *cel74a* gene from the fungal genome.

Phenotypic analysis was completed for both the parental and Δ*cel74a* strains grown on agar plates with different media compositions (Figure S3A). The Δ*cel74a* mutant strain showed no changes in growth or sporulation patterns when compared to the parental strain. In addition, we observed that both the parental and mutant strains produced similar rates of biomass production when grew in the presence of glycerol (Figure S3B). Taken together, these results suggest that deletion of *cel74a* not affected the vegetative growth of this fungus. Moreover, Δ*cel74a* exhibited no changes in CMC degradation assay when compared to the parental strain, suggesting that deletion of the xyloglucanase gene has no influence on the overall secretion of cellulolytic enzymes in *T. reesei* (Figure S3C). Interestingly, this result seems to be carbon-source dependent. The measurement of total proteins of the *T. reesei* secretome after sugarcane bagasse induction revealed that the mutant strain produces a greater amount of proteins when compared to the parental strain at 48, 72, and 96 h of cultivation (Figure S4). When compared, the results of CMC degradation and total protein production suggest that the mutant strain for xyloglucanase CEL74A secretes a higher concentration of proteins possibly involved in the degradation of the hemicellulose fraction of plant biomass.

### 2.4. Xylose, Glucose, and Galactose Release Is Altered by cel74a Deletion

**Figure 4:**
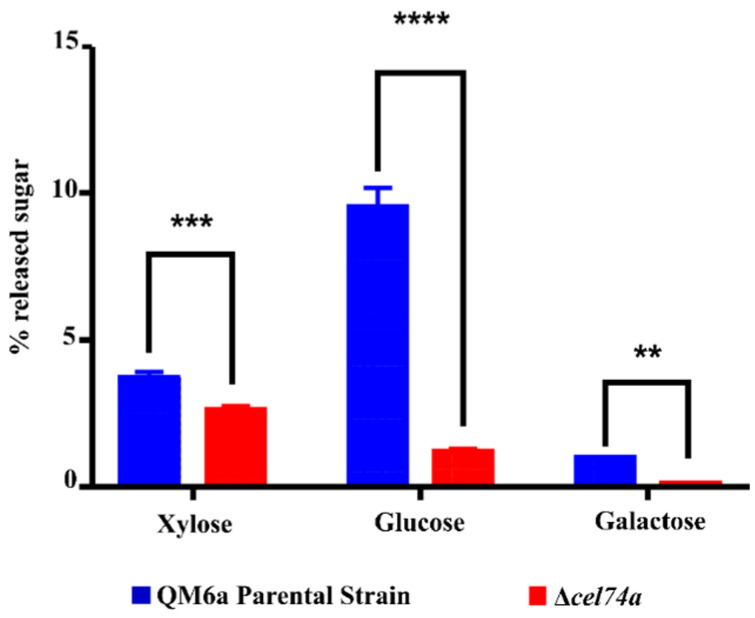
Released sugar profile after 72h of xyloglucan hydrolysis using QM6a and Δ*cel74a* supernatant after cultivation with SCB. **** (*p* < 0.0001), *** (*p* < 0.001), and ** (*p* < 0.01).

In order to test the role of CEL74A in xyloglucan hydrolysis, we performed HPLC analyses of the culture supernatants from both the Δ*cel74a* and parental strains grown in the presence of SCB for 72 h. The deletion of *cel74a* clearly altered the breakdown process of xyloglucan (Figure 4). The levels of xylose, glucose, and galactose were 1.5-fold, 8-fold, and 10-fold lower in the mutant than in the parental strain. Although the mutant strain secretes a greater amount of proteins after prolonged induction in sugarcane bagasse (Figure S4), this greater amount of proteins does not seem to guarantee full access to xyloglucan, which alters the percentage of release of sugars such as xylose, glucose, and galactose from xyloglucan. These results suggest that a functional CEL74A protein is necessary for efficient xyloglucan hydrolysis and that the absence of this enzyme might alter the pattern of the degradation of β-galactosidases, α-xylosidases, and β-glucosidases probably by reducing hemicellulose accessibility.

### 2.5. Absence of Xyloglucanase cel74a Affects Holocellulolytic Gene Expression in T. reesei

**Figure 5:**
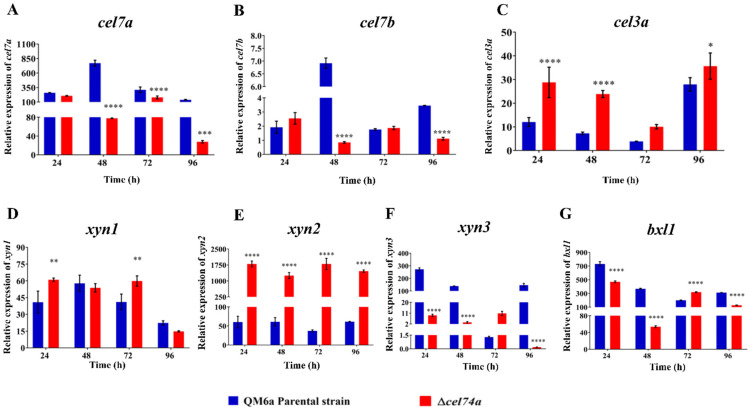
Expression profiles of holocellulolytic genes in the parental and Δ*cel74a* strains grown in the presence of SCB. (**A**) Relative expression of cellobiohydrolase *cel7a*, (**B**) Endo-β-1,4-glucanase *cel7b*, (**C**) β-glucosidase *cel3a*, (**D**) Endo-β-1,4-xylanase *xyn1*, (**E**) Endo-β-1,4-xylanase *xyn2*, (**F**) Endo-β-1,4-xylanase *xyn3,* and (**G**) β-xylosidase (*bxl1*). These results are based on three replicates of three independent experiments and are expressed as the mean ± standard deviation. **** (*p* < 0.0001), *** (*p* < 0.001), ** (*p* < 0.01), and * (*p* < 0.05).

The expression of the main *T. reesei* holocellulolytic genes (cellobiohydrolase *cel7a*, Endo-β-1,4-glucanase *cel7b*, and β-glucosidase *cel3a*) were analyzed following cultivation in the presence of SCB (Figure 5). As shown in Figure 5A, the deletion of xyloglucanase *cel74a* results in a decrease in the expression of cellobiohydrolase *cel7a* of approximately 10-, 2- and 5-fold after 48 h, 72 h, and 96 h of cultivation, respectively. Similarly, Endo-β-1,4-glucanase *cel7b* expression also decreased by eight- and threefold compared to the parental strain after 48 h and 96 h of cultivation, respectively (Figure 5B). In contrast, β-glucosidase *cel3a* was upregulated in response to *cel74a* deletion with its expression increasing at 24 h (2-fold), 48 h (3-fold), and 96 h (1.5-fold) of cultivation (Figure 5C). Our results suggest that *cel74a* affects cellulolytic gene expression in *T. reesei*.

We also evaluated the expression profile of the main hemicellulolytic genes of *T. reesei* in response to SCB cultivation. The Endo-β-1,4-xylanase gene *xyn1* increased by 1.5-fold at 24 h and 72 h of cultivation when compared to the parental strain (Figure 5D). Similarly, the expression of the Endo-β-1,4-xylanase gene *xyn2* was also upregulated when the mutant strain was grown in the presence of SCB. The expression of *xyn2* was upregulated at all cultivation times, being at least 20-fold higher than that of QM6a at each time point (Figure 5E). Interestingly, the expression of the Endo-β-1,4-xylanase gene *xyn3* was different from the other xylanase genes. The expression level of *xyn3* was downregulated in the mutant strain at 24 h, 48 h, and 96 h (Figure 5F), reaching values of 29-fold, 41-fold, and 813-fold less than the parental strain under the same conditions. Regarding this gene, the lowest expression level was detected after 96 h of SCB cultivation. Taken together, our results suggest that xyloglucanase CEL74A may differentially regulate the expression of hemicellulase genes via a distinct and still unknown molecular mechanism in response to SCB.

Finally, we went on to demonstrate that the expression of the β-xylosidase gene *bxl1* was regulated in the absence of *cel74a*. The expression level of *bxl1* was downregulated in the mutant strain compared to the parental strain at 24 h, 48 h, and 96 h induction times, being the lowest expression level of *bxl1* gene detected after 48 h of cultivation with SCB (Figure 5G). Additionally, our results shown a slight increase in *bxl1* expression at 72 h of induction in the presence of SCB.

When compared the results of expression of *xyn3* and *bxl1*, we can see that these genes have a tendency to decrease their expression in the Δ*cel74a* strain. Although the lowest levels of expression for these genes are detected at different cultivation times (96h for *xyn3* and 48 h for *bxl1*), together our results suggest that *xyn3* and *bxl1* have a similar pattern of genic regulation in *T. reesei* in the absence of *cel74a*.

### 2.6. Absence of Xyloglucanase cel74a Affects Holocellulase Activities in T. reesei

**Figure 6:**
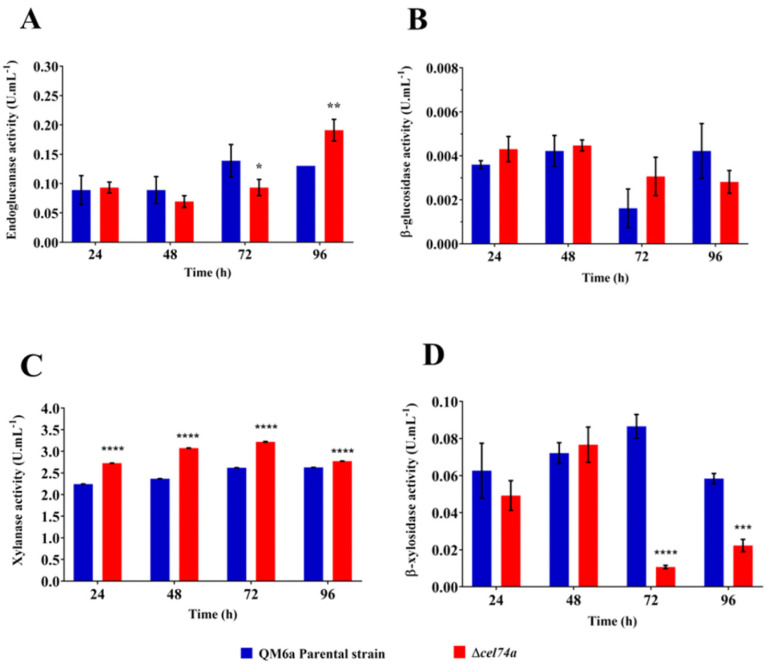
Holocellulolytic activities of QM6a and Δ*cel74a* grown in SCB. (**A**) Endoglucanase (CMCase), (**B**) β-glucosidase, (**C**) Xylanase, and (**D**) β-xylosidase activity in the culture supernatant of *T. reesei*. QM6a or Δ*cel74a* strains grown in the presence of SCB. **** (*p* < 0.0001), *** (*p* < 0.001), ** (*p* < 0.01), and * (*p* < 0.05).

To determine whether increased/decreased holocellulolytic gene expression was followed by changes in cellulase/hemicellulase activity in Δ*cel74a* we completed activity assays for endoglucanase (CMCase), β-glucosidase, xylanase, β-xylosidase, β-galactosidase, β-1,3-glucanase, and xyloglucanase. The endoglucanase activity profile of Δ*cel74a* was similar to that of the QM6a during SCB cultivation (Figure 6A) although there was a slight reduction in CMCase activity (endo-β-1,4-glucanase) in the mutant strain at 72 h of cultivation in the presence of SCB. Interestingly, there was an increase in activity at 96 h of cultivation in SCB and although the transcriptional analysis showed that there was a reduction in *cel7b* (Endo-β-1,4-glucanase) expression in the mutant strain, this reduction was not reflected in its endoglucanase activity. Similarly, even with the increase in β-glucosidase *cel3a* expression after cultivation in SCB (Figure 5C), there was no increase in enzymatic activity in the presence of this carbon source (Figure 6B).

The Δ*cel74a* xylanase activity was increased in comparison to the parental strain after cultivation in SCB (Figure 6C). This result agrees with the increased expression of both *xyn1* and *xyn2* observed in the qPCR analyses (Figure 5D,E). Conversely, the reduced *bxl1* expression did not translated to reduced β-xylosidase activity in the Δ*cel74a* strain. Figure 6D demonstrates that β-xylosidase activity was downregulated by eight- and two-fold, respectively, after 72 h and 96 h of cultivation in SCB. Therefore, our results suggest that *T. reesei* CEL74A might be important in the regulation of both xylanase and β-xylosidase activity.

**Figure 7:**
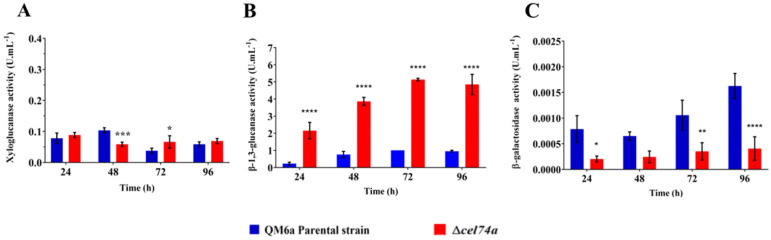
Holocellulolytic activities in QM6a and Δ*cel74a* grown on SCB. (**A**) Xyloglucanase, (**B**) β-1,3-glucanase (laminarinase), (**C**) β-galactosidase activity in the *T. reesei* supernatants. **** (*p* < 0.0001), *** (*p* < 0.001), ** (*p* < 0.01), and * (*p* < 0.05).

Different enzymes including xyloglucanases, β-galactosidases, β-xylosidases, and β-glycosidases act sequentially to promote xyloglucan hydrolysis. To evaluate the dynamic of xyloglucan hydrolysis, the enzymatic activities were assayed in the Δ*cel74a* strain. Curiously, the xyloglucanase activity profile of the mutant strain was similar to that of the parental strain at 24 h and 96 h of cultivation, exhibiting a slight reduction at 48 h and an increase after 72 h of cultivation (Figure 7A). Since we expected that the xyloglucanase activity in the mutant strain would be completely abolished this result suggests that xyloglucan is probably not a specific substrate for xyloglucanase activity.

Since xyloglucan is not a specific substrate for xyloglucanase, we also assayed the β-1,3-glucanase and β-galactosidase activities of the Δ*cel74a* strain. β-1,3-glucanase exhibited an increase in activity of 10-, 5-, 5-, and 5-fold at 24, 48, 72, and 96 h, respectively, when compared to the parental strain (Figure 7B). In contrast, β-galactosidase activity was reduced in the Δ*cel74a* strain when compared to the parental strain at all time points (Figure 7C). These results suggest that laminarinase (β-1,3-glucanase) activity may be regulated by the *cel74a* gene, and that this may be a compensatory mechanism in *T. reesei* allowing for the maintained xyloglucanase activity in the mutant strain. This suggests that there may be some redundancy in this pathway and that endo-β-1,3-glucanase activity is central to this phenotype.

## 3. Discussion

Hemicellulose hydrolysis produces a wide variety of sugars including xylose, arabinose, mannose, glucose, and galactose monomers. In the natural environment, plant biomass is degraded by a diverse selection of microorganisms that promote the coordinated degradation of lignocellulolytic material via the production of specific hydrolytic enzymes [35,36,37,38,39]. Given this cellulase-producing microorganisms, especially filamentous fungi, have been the focus of several studies designed to uncover new enzymes and elucidate the underlying mechanisms regulating enzyme production and activity [18,30,40,41].

The structural architecture of the SCB includes a wide range of polymers including arabinoxylan, xyloglucan, mixed bonded β-glycan and pectin making it an ideal substrate for evaluating microbial processes and the generation of value-added products [18,42]. In addition, the enormous complexity of the plant cell wall is a barrier that must be overcome if we ever plan on using these agroindustrial waste products in industrial processes [43]. *T. reesei* breaks down SCB via the coordinated effects of several enzymes including endopolygalacturonases, pectin-methyl-esterases, α-arabinofuranosidases, β-galactosidases, lichenases, feruloyl esterases, acetyl esterases, endo-β-xylanases, xyloglucanases, α-xylosidases, and β-galactosidases [13]. Borin et al. [18] found that *T. reesei* degrades the cellulose-xyloglucan network more efficiently than other microbes because of its ability to degrade the cellulose microfibrils. Likewise, Daas et al. [44] showed that *Geobacillus thermodenitrificans* T12 secretes an endo-xylanase (*GtXynA*), which is the key regulator initiating hemicellulose degradation.

These findings suggest that xyloglucanases are key enzymes in the regulation of other holocellulolytic enzymes, making them a key factor in the industrial degradation of lignocellulolytic biomass [45]. These enzymes have a dual role, via either their enzymatic activity, that attacks the structure of xyloglucan or via their release of specific sugar residues that induce specific signaling pathways upregulating the expression of other *T. reesei* cellulase enzymes [19]. Here, we constructed and characterized a null mutant strain for *T. reesei* xyloglucanase CEL74A. Additionally, several biochemical and molecular parameters were analyzed in order to understand the role of xyloglucanase CEL74A in the regulation of the expression of holocellulolytic genes in response to SCB.

Our results demonstrate that the deletion of xyloglucanase *cel74a* significantly affects the expression of holocellulolytic genes during cultivation in SCB. However, despite the reduction in the expression of cellobiohydrolase *cel7a* and Endo-β-1,4-glucanase *cel7b,* and an increase in the expression of β-glucosidase *cel3a*, there was no difference in the cellulolytic activity of the mutant and parental strains. The Δ*cel74a* strain produced more Endo-β-1,4-xylanases *xyn1* and *xyn2* and increased xylanolytic activity when compared to the parental strain. Together, our results suggest that the Δ*cel74a* strain is able to more effectively utilize the xylan fraction present in SCB as an energy source instead of the cellulosic fraction. This may indicate that there is a greater availability of xylose and xylo-oligosaccharides in the medium originating from the hydrolysis of xylan present in SCB. The reduction in the expression of the Endo-β-1,4-xylanase *xyn3* supports this hypothesis, since unlike *xyn1* and *xyn2*, which are induced in the presence of these substrates, the *xyn3* gene does not undergo induction under these conditions and is only expressed in the presence of cellulases-inducing substrates [46].

Our results also demonstrate that *T. reesei* CEL74A activity is modulated by calcium. In the QM6a strain, the presence of calcium increased xyloglucanase activity, while addition of EDTA reduced xyloglucanase activity. This suggests that CEL74A function is dependent on the activity of the calcium binding site and that this site regulates xyloglucanase activity in *T. reesei*. Calcium dependent signaling pathways have been implicated in the regulation of holocellulase expression in *T. reesei* in previous studies. Mach et al. [47] showed that calmodulin, a key protein in calcium signaling, is essential for the regulation of xylanase expression. Likewise, the interaction of the calcium binding proteins NCS (neuronal calcium sensor-like), phosphodiesterase, and ENVOY (a protein with PAS/LOV domain) have all been linked to the regulation of G protein signaling and its induction of cellulase gene expression [48]. Recently, Martins-Santana et al. [49] showed that the deletion of CRZ1 (calcineurin-responsive zinc finger 1) affects the expression of cellulases in *T. reesei* and that Ca^2+^ acts synergistically with CRZ1 to regulate gene expression in these fungi. However, Ca^2+^ was also shown to exhibit a *CRZ1*-independent regulatory role in *T. reesei* gene regulation. Taken together these results highlight the role of Ca^2+^ as a major regulator of holocellulase transcription.

Given the stability of overall cellulase activity, we can assume that the absence of xyloglucanase CEL74A in the parental strain may induce an increase in CEL12A activity, suggesting a compensatory mechanism for efficient xyloglucan breakdown. Another hypothesis is that probably there may be an increase in the secretion of an endo-1,3-(4)-β-glucanase, which has some small activity against xyloglucan, in the Δ*cel74a* which may compensate for its absence. It is known that enzymes of this class may exhibit activity against a variety of substrates including β-glucan, lichenan, and laminarin amongst others, which could explain the increase in laminarinase activity (endo-β-1,3-glucanase) observed in this strain in the presence of SCB [50,51,52,53].

The reduction in the release of galactose, glucose, and xylose from xyloglucan might be explained by the absence of the exo/endoglucanases activity of in the Δ*cel74a* strain reducing the availability of these smaller oligosaccharides and reducing their downstream induction effects ultimately reducing the activity of other enzymes, such as β-galactosidases and α-xylosidases, in the degradation of these cellulosic substrates. This hypothesis is supported by the concurrent reduction in β-galactosidase activity. Furthermore, even that Δ*cel74a* strain is able to secrete a high concentration of proteins in their secretome when compared to QM6A, this ability seems to have no effects on xyloglucan degradation without a functional CEL74A.

The expression of holocellulolytic genes in *T. reesei* is differentially regulated in response to the composition of the complex carbon sources in the growth medium. SCB is one such complex carbon source and thus we see a varied and regulated response to its addition to the media. Once growing in this carbon source, *T. reesei* may activate specific signaling pathways to coordinate its transcriptional response and induce the most effective combination of holocellulase genes. When taken together our results demonstrate that efficient xyloglucan degradation is dependent on CEL74A which is critical to the optimal performance of various other enzymes involved in this process. Thus, further experiments are necessary to understand the cell signaling events involved in the use of this substrate in *T. reesei*. In addition, our findings represent a large expansion in our understanding of the coordinated regulation of these enzymes and may serve as the basis for developing a deeper understanding of the protein regulation and signaling pathways involved in biomass degradation. Finally, our results suggest for the first time the specific role of xyloglucanase CEL74A in the regulation of gene expression and enzymatic activity of holocellulases in *T. reesei*. However, additional experiments should be carried out for the functional characterization of CEL74A.

## 4. Materials and Methods

### 4.1. Strains and Culture Conditions

*T. reesei* strain QM6aΔ*tmus53*Δ*pyr4* [34] was obtained from the Institute of Chemical Engineering & Technical Biosciences at the Vienna University of Technology (TU Vienna), Austria. The strain was maintained at 4 °C on MEX medium (malt extract 3% (*w/v*) and agar–agar 2% (*w/v*)), and in the case of the *pyr4* deletion strain supplemented with 5 mM uridine. The *T. reesei* QM6aΔ*tmus53*Δ*pyr4* parental and Δ*cel74a* mutant strains were grown on MEX medium at 30 °C for 7–10 days until complete sporulation.

### 4.2. Phenotypic Characterization

For the phenotypic assays, the parental and mutant strains were inoculated on Mandels–Andreotti medium containing 1% of one of the following carbon sources: glucose, xylose, mannose, lactose, maltose, arabinose, or microcrystalline cellulose (Avicel) on agar plates. For the Congo red assay, the CMC (Carboxymethyl cellulose) containing parental and mutant strain agar plates were stained with 1 mg/mL Congo red solution for about 15 min and de-stained using three 1 M sodium chloride washes for 15 min each. For the biomass production assay, 10^6^ cells/mL were grown on Mandels–Andreotti medium supplemented with 1% (*w/v*) glycerol for 24 h and then the mycelia were collected, dried, and weighed for comparison. All experiments were carried out in biological triplicate.

### 4.3. Vector Construction for Gene Deletion

Phusion High-Fidelity DNA Polymerase (Thermo Scientific, Waltham, MA, USA) was used to produce all the sequences used in the cloning experiments. All primers used in this study are listed in Table S1. PCR (Polymerase chain reaction) products were cloned into pJET1.2/blunt (Thermo Scientific, Waltham, MA, USA) for use downstream. To construct the *cel74a* deletion plasmid (pCD-Δcel74a), the 5′ (≅1.1 Kb) and 3′ (≅1.2 kb) regions of the *cel74a* gene were amplified by PCR using *T. reesei* QM6a DNA as template and primers *cel74a*_5fwd-*Hind*III and *cel74a*_5rev-*Xba*I or *cel74a*_3fwd-*Bgl*II and *cel74a*_3rev-*Not*I, respectively (Table S1, Figure S1). These flanking regions were then consecutively cloned into the pJET-*pyr4* vector [54] and then digested using the appropriate restriction enzymes and cloned into the final deletion vector. Finally, pCD-Δ*cel74a* was linearized using *NotI* and then purified for use in the *T. reesei* transformations (Figure S1).

### 4.4. Transformation of T. reesei

The protoplast transformations of *T. reesei* QM6aΔ*tmus53*Δ*pyr4* were carried out as previously described [55] and a total of 40 µg of linearized pCD*-Δcel74a* was used in each assay. After transformation, the plates were incubated at 30 °C for 3–4 days until colonies were visible. Candidates were then subjected to three rounds of homokaryon selection using spore streak outs on minimal medium selection plates (1 g/L MgSO_4_·7H_2_O, 10 g/L 1% KH_2_PO_4_, 6 g/L (NH_4_)2SO_4_, 3 g/L trisodium citrate·2H_2_O, 10 g/L glucose, 20 mL/L 50X trace elements solution (0.25 g/L FeSO_4_·7H_2_O, 0.07 g/L ZnSO_4_·2H_2_O, 0.1 g/L CoCl_2_·6H_2_O, 0.085 g/L MnSO_4_·H_2_O), 0.1% Triton X-100, 2% (*w/v*) noble agar lacking uridine) (all from Sigma Aldrich, St. Louis, MO, USA) until stable homokaryotic strains were obtained. Cassette integration was verified by PCR using the *Pyr4*_5′rev or *Pyr4*_3′fwd (inside the selectable marker gene *pyr*4) primers and the gene-specific *cel74a*_SC_F or *cel74a*_SC_R (outside the transformation cassette) primers (Table S1). The expression profiles of the *cel74a* gene were also analyzed by RT-qPCR.

### 4.5. Gene Expression Analysis

For the gene expression assays, a spore resuspension solution containing approximately 10^6^ cells/mL of *T. reesei* QM6aΔ*tmus53*Δ*pyr4* and Δ*cel74a* strains were inoculated into 40 mL of Mandels–Andreotti medium supplemented with 1% (*w/v*) SCB, 1% (*w/v*) glycerol, 2% (*w/v*) glucose, or 1% (*w/v*) microcrystalline cellulose as the sole carbon source at 30 °C on an orbital shaker (200 rpm). The preparation of exploded sugarcane bagasse was carried out as previously described [31,56]. The parental and Δ*cel74a* strains were grown in 1% (*w/v*) glycerol for 24 h and then transferred to medium containing SCB, glucose, or microcrystalline cellulose (Avicel) in all experiments. The resulting mycelia were collected by filtration, frozen in liquid nitrogen, and stored at −80 °C for RNA extraction. The supernatants were harvested and used to determine enzyme activities, and xyloglucan hydrolysis profile.

### 4.6. RNA Extraction and Transcript Analyis Using Quantitative PCR (RT-qPCR)

Total RNA was isolated from mycelia using TRIZOL reagent (Thermo Fisher Scientific, Waltham, MA) according to the manufacturer’s instructions and treated with DNase I (Thermo Scientific, Waltham, MA, USA) to remove genomic DNA contamination. cDNA (complementary DNA) synthesis was then carried out using the Maxima™ First Strand cDNA Synthesis kit (Thermo Scientific, Waltham, MA, USA) according to the manufacturer’s instructions then diluted 1:50 and analyzed using the CFX96™ Real-Time PCR Detection System (Bio-Rad Laboratories, Hercules, California, CA, USA) and the SsoFast™ EvaGreen^®^ Supermix (Bio-Rad Laboratories, Hercules, California, CA, USA), in accordance with the manufacturer’s instructions. Each reaction (10 µL) contained 5 µL of SsoFastTM EvaGreen^®^ Supermix (Bio-Rad Laboratories, Hercules, California, CA, USA), forward and reverse primers (500 nm each; Table S1), cDNA template, and nuclease-free water. PCR cycling conditions were as follows: 10 min at 95 °C, followed by 40 cycles of 10 s at 95 °C and 30 s at 60 °C. Melt analysis used a ramp of 60–95 °C at a rate of 0.5 °C/10 s to evaluate primer dimers and nonspecific amplification. The *Sar1* GTPase transcript was used as an internal reference to normalize the amount of total RNA present in each reaction [57]. In both the SCB and Avicel expression analyses, gene expression levels were calculated using the 2^−ΔΔCT^ method [58], relative to the parental QM6a strain grown under non-inducing conditions (glycerol) for 24 h [59]. For glucose, gene expression levels were calculated using the 2^−ΔCT^ method [58], relative to the transcript levels of *Sar1* GTPase.

### 4.7. Enzyme Activity Assays

The activities of the CMCase, β-1,3-glucanase, xylanase, and xyloglucanase enzymes were evaluated using 3,5-dinitrosalicylic acid (DNS) and monitored for colorimetric change at 540 nm [31,60]. The CMCase activity was determined using 30 μL of 1% carboxymethylcellulose (CMC) (Sigma Aldrich, St. Louis, MO, USA) in sodium acetate buffer (50 mM, pH 4.8) and 30 μL of enzyme (culture supernatant). After a 30 min incubation at 50 °C, 60 μL of DNS was added, followed by heating at 95 °C for 5 min to allow color development. Then, the samples were transferred to a flat-bottomed microplate and absorbance at 540 nm was read. The enzyme unit (U) was defined as the amount of enzyme needed to release 1 μmol of reducing sugar per minute. To determine the β-1,3-glucanase activity, 20 μL of 0.75% laminarin (Sigma Aldrich, St. Louis, MO, USA) in sodium acetate buffer (50 mM, pH 5.0) and 10 μL of enzyme were incubated for at 50 °C for 10 min and then evaluated using DNS as described above. Xylanase activity was measured by mixing 25 μL of 1% xylan beechwood (Sigma Aldrich, St. Louis, MO, USA) in sodium acetate buffer (100 mM, pH 5.0) and 10 μL of enzyme and incubating at 50 °C for 30 min. Color was developed using 75 μL of DNS and the conditions described above.

Xyloglucanase activity was assayed using 50 μL of 1% xyloglucan obtained from *Hymenaea courbaril var. courbaril* seeds, as previously described [61] in sodium acetate buffer (100 mM, pH 5.0) and 50 μL of enzyme (culture supernatant). After a 30 min incubation at 50 °C, 100 μL of DNS was added, followed by heating at 95 °C for 5 min to allow color development. Then, the samples were transferred to a flat-bottomed microplate and absorbance at 540 nm was read. Then to evaluate the impact of Ca^2+^ on xyloglucanase activity we repeated the relevant assays in the presence of 10 mM CaCl_2_ or 2.5 mM EDTA, respectively.

Finally, β-glucosidase, β-galactosidase, and β-xylosidase activities were determined using p-nitrophenyl-derived substrates: p-nitrophenyl β-d-glucopyranoside (pNPGluc) (5 mM), p-nitrophenyl α-d-galactophyranoside (pNPGal) (5 mM), and p-nitrophenyl β-D-xylopyranoside (pNPXyl) (5 mM) (Sigma Aldrich, St. Louis, MO, USA). The β-glucosidase and β-xilosidases reactions were carried out in a microplate assay format and the assay mixture contained 10 μL of the enzyme solution, 40 μL of the p-nitrophenyl-derived solution, and 50 μL (β-xilosidases) or 100 μL (β-galactosidase and β-xilosidases) of the relevant enzyme in 50 mM sodium acetate buffer. The mixtures were buffered at pH 5.5 (β-glucosidase) or pH 5.0 (β-galactosidase and β-xilosidases) and after 15 min at 50 °C the reactions were stopped by adding 100 μL of 1M Na_2_CO_3_. The amount of p-nitrophenol was determined using a spectrophotometer at 405 nm. One unit of enzyme activity was defined as the amount of enzyme necessary to release 1 μmol of p-nitrophenol per minute. All enzyme assays were completed in triplicate for each sample.

### 4.8. Total Protein Quantification

In order to determine the total protein concentration of *T. reesei* secretome, the resulting culture supernatant of sugarcane bagasse induction was collected and employed for protein quantification. The protein concentration was determined using the kit Bio-Rad Protein Assay (Bio-Rad Laboratories, Hercules, California, CA, USA), based on the Bradford method. All experiments were performed in three biological replicates.

### 4.9. Xyloglucan Sugar Release Profile of T. reesei Δcel74a

Sugar hydrolysis was carried out as previously described by [57] with few modifications. To analyze the hydrolysis profile of xyloglucan, we mixed 7 mL of 1% xyloglucan in 100 mM sodium acetate buffer (pH 5.0) and 7 mL of *T. reesei* (parental or Δ*cel74a* strain) culture supernatant (grown in SCB for 48 h) at 50 °C on an orbital shaker (180 rpm). Then 1 mL aliquots of these hydrolysis reactions were collected every 24 h over the next 72 h and filtered through a 0.22 μm filter and then evaluated for monomeric sugar content (glucose, xylose, and galactose) using high performance liquid chromatography (HPLC) (YL9100 HPLC System, Allcrom—Young Lin) with a series refractive index detector (YL9170) at 40 °C. The samples were applied to a REZEX Roa column (Phenomenex, Torrance, California, CA, USA) (300 × 7.8 mm) at 85 °C and eluted in 0.005 M sulfuric acid at 0.5 mL/min. The results were processed using the Clarity software with sugar standards as a reference. All tests were performed in triplicate.

### 4.10. D Structure Prediction and Phylogenetic Analysis

I-TASSER was accessed from an online platform (https://zhanglab.dcmb.med.umich.edu/I-TASSER/, version 5.1, University of Michigan, Ann Arbor, Michigan, MI, USA) and used to determine the 3D structure of the xyloglucanase protein. To do this a FASTA file containing the amino-acid sequence for the xyloglucanase protein was obtained from the UniProt database (*T. reesei* xyloglucanase *cel74a*, protein ID: 49081) and submitted to the I-TASSER online server. Then the following templates from the PDB online database were used to predict the 3D structure*: 4lgnA* from *Neurospora crassa* [62], *5fkqA* from *Clostridium thermophilus* [63] and *2ebsA* from *Aspergillus nidulans* [64]. The structure modeling approach used by this software is based on sequence alignment to a protein template, which is identified by LOMETS, a method that selects the top 10 alignments in the PDB library. The unaligned regions of the sequence are built using ab initio folding which uses the lowest-free energy states and minimized steric interference as identified by SPICKER and TM-align, respectively, to produce a probable structure. The final models are then evaluated at the atomic level using REMO, which optimizes the hydrogen-bonding profile. The I-TASSER online software also allows the prediction of the biological function of the protein as it compares its predicted structural models to varies protein libraries with defined biological functions [65,66,67]. All predictions along with the ligands provided by the I-TASSER platform were visualized and configured using the PyMOL (https://pymol.org/2/, version 1.8.6.0, Schrödinger LLC, New York, NY, USA).

The selection of the most appropriate model relies on both C-score and TM-score confidence values. The first estimates the quality of the prediction, which means that the higher the value the higher the confidence. The latter measures the structural similarity between the predicted model and the template used for its construction, with values of >0.5 indicating that the structure is likely to be in the correct topology, and <0.17 likely the result of random similarity. RMSD (Root-Mean-Square Deviation) can also be used to evaluate the similarity between the predicted structure and the template, although it may produce an inaccurate evaluation even if the model is correct if there are a large number of local errors. These three scores are highly correlated, and their combined use is usually representative. Maximum-parsimony phylogenetic trees for the conserved xyloglucanase genes were generated using iTOL [32,33].

### 4.11. Statistical Analysis

All statistical significance was evaluated using analysis of variance (ANOVA) and Bonferroni pairwise comparisons (*p* ≤ 0.05) in GraphPad Prism v. 7.0 Software.

## Data Availability

The data presented in this study are available on request from the corresponding author.

## Supplementary Materials

The following are available online at https://www.mdpi.com/article/10.3390/ijms22094545/s1, Figure S1: Amplification of fragments of *T. reesei cel74a*. The 5′ (promoter) and 3′ (terminator) regions of the *cel74a* gene were amplified, confirmed*,* and cloning was performed in the vector pJET1.2/blunt. (A) PCRs from the 5′ and 3′ regions of the *cel74a* gene. (B) Digestion of the pJET1.2/blunt vector with *Hind*III and *Xba*I enzymes to verify the cloning of the 5′ region of *cel74a*. (C) Digestion of the pJET1.2/blunt vector using *Not*I and *Bgl*II enzymes to verify the identity of the 3′ *cel74a* sequence. (D) Digestion of the plasmid pCD-*pyr4* containing the *5′ cel74a* sequence. (E) Digestion of the pCD-Δ*cel74a* plasmid used in fungal transformation. (F) Schematic representation of the pCD-Δ*cel74a* plasmid. MM: molecular weight marker (O’GeneRuler ™ 1 Kb DNA Ladder, Thermo Fisher Scientific), Figure S2: PCR and qRT-PCR used to identify the mutant strain. (A) Schematic describing the partial deletion of *cel74a* in the parental strain QM6aΔ*tmus5*3Δ*pyr4* (Δ*tmus53*Δ*pyr4*) by homologous recombination using plasmid pCD-Δ*cel74a* to produce the *cel74a* deletion strain (Δ*tmus53*Δ*cel74a*). (B) PCR amplification of the molecular marker *(pyr4*) during the validation of *the Δcel74a* mutant strain. (C) PCR amplification of the coding region of *cel74a* validating the creation of the Δ*cel74a* mutant strain. QM6a: positive control. (D) qRT-PCR analysis of *cel74a* used to validate *cel74a* deletion. cDNA from the QM6a parental strain was used as a positive control. MM: molecular weight marker (O’GeneRuler ™ 1 Kb DNA Ladder, Thermo Fisher Scientific)**, Figure S3**: Phenotypic characterization of the Δ*cel74a* mutant strain. (A) Growth of the *T. reesei* parent strain and Δ*cel74a* on plates with different carbon sources after seven days of cultivation. (B) Growth of *T. reesei* QM6a and Δ*cel74a* strains using glycerol as a carbon source. Error bars represent the data from three biological replicates. No significant differences in growth were observed between the Δ*cel74a* mutant strain and the parental QM6a strain (*p* ≤ 0.05). (C) CMC degradation halos in the QM6a and Δ*cel74a* strains. CMC degradation halos were measured after 4 days of growth from three independent experiments completed in biological triplicate. No significant differences in CMC degradation were observed (*p* ≤ 0.05), Figure S4. Total proteins production of the *T. reesei* (parental and Δ*cel74a* strain) secretome after sugarcane bagasse induction. Significant differences in total protein concentration were observed between the ∆*cel74a* mutant strain and the parental QM6a strain at 48 h (*p* ≤ 0.05), 72 h and 96 h (*p* < 0.0001). No significant differences in total protein concentration were observed after 24 h of cultivation on sugarcane bagasse. Error bars represent the data from three independent experiments completed in biological triplicate. Table S1: Primer sequences used in this study.
