# Supplementary material for: Molecular Characterization of Xyloglucanase cel74a from Trichoderma reesei
Source: Int J Mol Sci. 2021 Apr 27;22(9):4545. doi: 10.3390/ijms22094545 (PMC8123685; doi:10.3390/ijms22094545)
Supplement: Supplementary file 1 [file ijms-22-04545-s001.zip › ijms-1200573-supplementary.pdf]

International Journal of *Molecular Sciences*

MDPI

Molecular characterization of xyloglucanase cel74a from *Trichoderma reesei*

Douglas Christian Borges Lopes[1], Cláudia Batista Carraro[1], Roberto Nascimento Silva[1], Renato Graciano de Paula[1,2*]

[1] Molecular Biotechnology Laboratory, Department of Biochemistry and Immunology, Ribeirao Preto Medical School (FMRP), University of Sao Paulo, Ribeirao Preto, SP, 14049-900, Brazil ; douglas.chr.94@gmail.com, claudiacarraro@usp.com, rsilva@fmrp.usp.br

[2] Department of Physiological Sciences, Health Sciences Centre, Federal University of Espirito Santo, Vitoria, ES, 29047-105, Brazil ; renato.paula@ufes.br

* Correspondence: rsilva@fmrp.usp.br, Molecular Biotechnology Laboratory, Department of Biochemistry and Immunology, Ribeirao Preto Medical School (FMRP), University of Sao Paulo, Ribeirao Preto, SP, 14049-900, Brazil

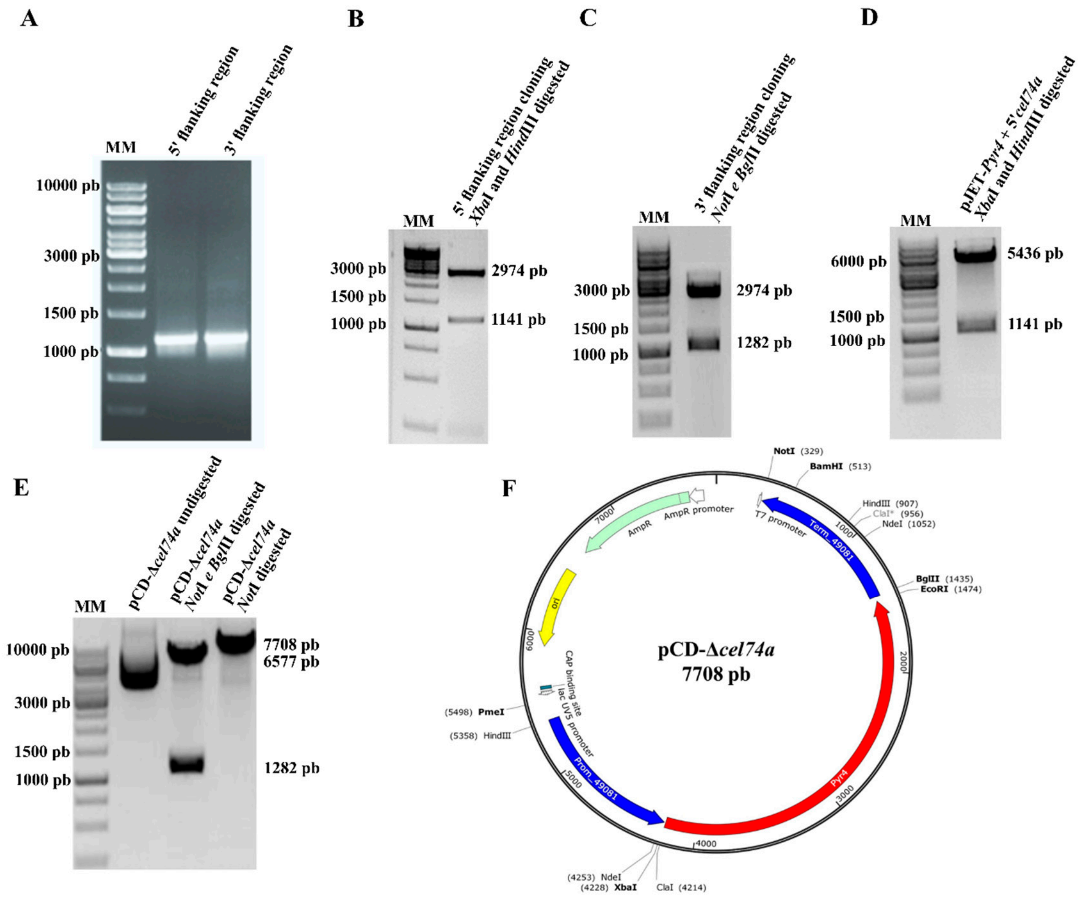

Figure S1. Amplification of fragments of *T. reesei* cel74a. The 5′ (promoter) and 3′ (terminator) regions of the *cel74a* gene were amplified, confirmed, and cloning was performed in the vector pJET1.2/blunt. (A) PCRs from the 5′ and 3′ regions of the *cel74a* gene. (B) Digestion of the pJET1.2/blunt vector with *Hind*III and *Xba*I enzymes to verify the cloning of the 5′ region of *cel74a*. (C) Digestion of the pJET1.2/blunt vector using *Not*I and *Bgl*II enzymes to verify the identity of the 3′ *cel74a* sequence. (D) Digestion of the plasmid pCD-*pyr4* containing the *5′ cel74a* sequence. (E) Digestion of the pCD-Δ*cel74a* plasmid used in fungal transformation. (F) Schematic representation of the pCD-Δ*cel74a* plasmid. MM: molecular weight marker (O'GeneRuler ™ 1 Kb DNA Ladder, Thermo Fisher Scientific).

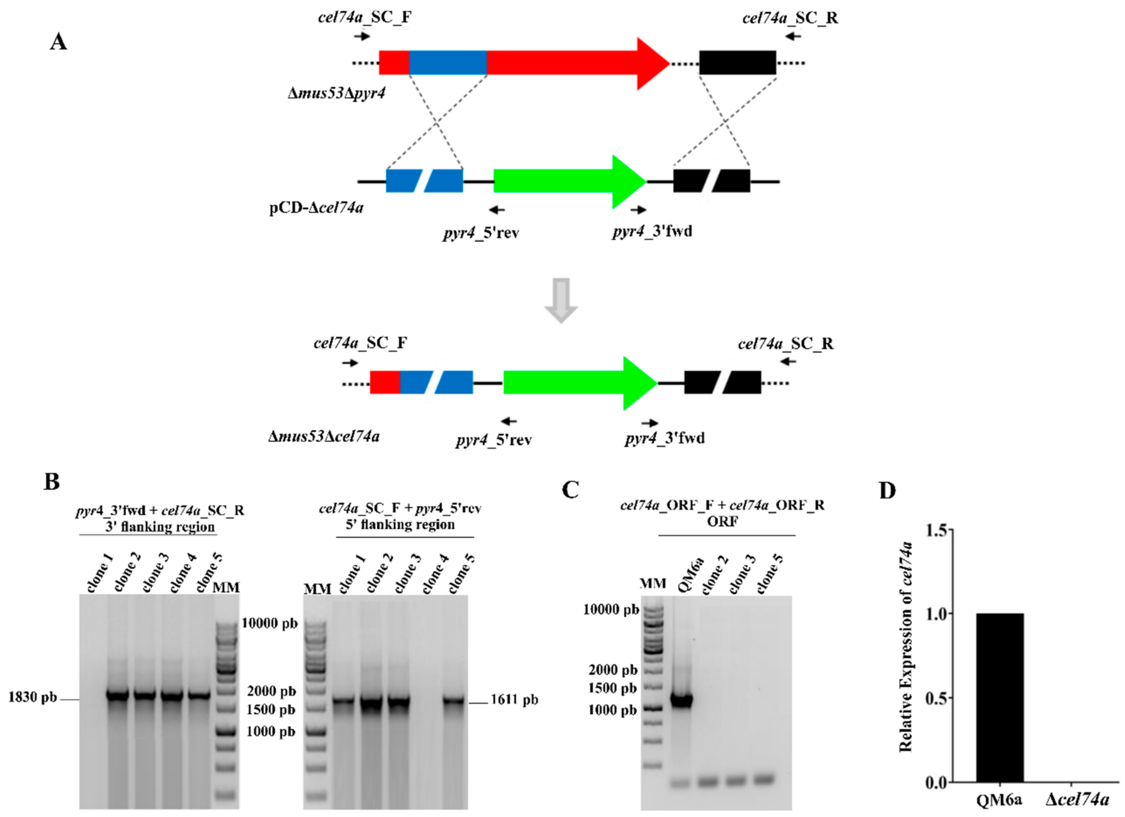

Figure S2. PCR and qRT-PCR used to identify the mutant strain. (A) Schematic describing the partial deletion of *cel74a* in the parental strain QM6aΔ*tmus53*Δ*pyr4* (Δ*tmus53*Δ*pyr4*) by homologous recombination using plasmid pCD-Δ*cel74a* to produce the *cel74a* deletion strain (Δ*tmus53*Δ*cel74a*). (B) PCR amplification of the molecular marker *(pyr4*) during the validation of *the Δcel74a* mutant strain. (C) PCR amplification of the coding region of *cel74a* validating the creation of the *Δcel74a* mutant strain. QM6a: positive control. (D) qRT-PCR analysis of *cel74a* used to validate *cel74a* deletion. cDNA from the QM6a parental strain was used as a positive control. MM: molecular weight marker (O'GeneRuler ™ 1 Kb DNA Ladder, Thermo Fisher Scientific).

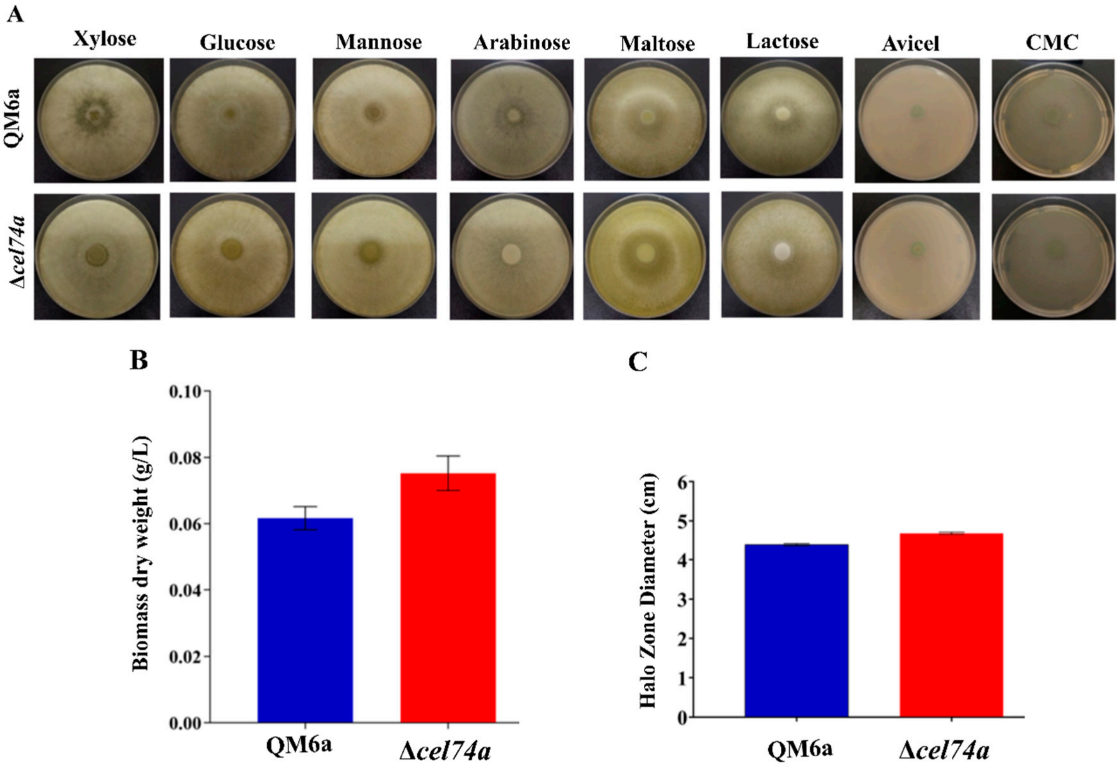

Figure S3. Phenotypic characterization of *the Δcel74a* mutant strain. (A) Growth of the *T. reesei* parent strain and Δ*cel74a* on plates with different carbon sources after seven days of cultivation. (B) Biomass dry weight of growth of *T. reesei* QM6a and Δ*cel74a* strains using glycerol as a carbon source. Error bars represent the data from three biological replicates. No significant differences in growth were observed between the *Δcel74a* mutant strain and the parental QM6a strain (p ≤ 0,05). (C) CMC degradation halos in the QM6a and Δ*cel74a* strains. CMC degradation halos were measured after 4 days of growth from three independent experiments completed in biological triplicate. No significant differences in CMC degradation were observed (p ≤ 0,05).

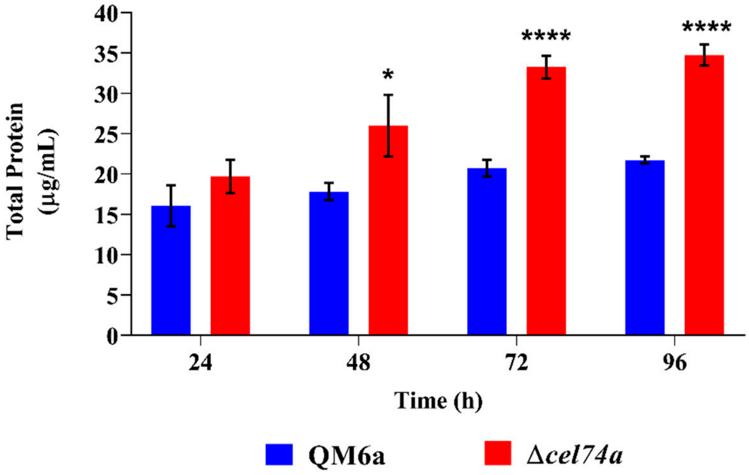

Figure S4. Total proteins production of the *T. reesei* (parental and Δ*cel74a* strain) secretome after sugarcane bagasse induction. Significant differences in total protein concentration were observed between the *Δcel74a* mutant strain and the parental QM6a strain at 48 hours (p≤0,05), 72 hours and 96 hours (p<0,0001). No significant differences in total protein concentration were observed after 24 hours of cultivation on sugarcane bagasse. Error bars represent the data from three independent experiments completed in biological triplicate.

Table S1. Primer sequences used in this study.

Primer	Sequence (5′ – 3′)	Amplicon	Amp

Copyright: © 2021 by the authors. Licensee MDPI, Basel, Switzerland. This article is an open access article distributed under the terms and conditions of the Creative Commons Attribution (CC BY) license

(http://creativecommons.org/licenses/by/4.0/).			
cel74a_5fwd-*Hind*III	GGGGGAAGCTTCACAGAGATACGCTTTGCAGGG	1141 (bp)	5′-flan
cel74a_5rev-*Xba*I	GGGGGTCTAGATCTGTTCGTGCATATGCTACGCCT		
*cel74a*_3fwd-*Bgl*II	GGGGGAGATCTTCATTATGCTCAGTGCGGAGG	1282 (bp)	3′-flan
*cel74a*_3rev-*Not*I	GGGGCGGCCGCGTCTTCATCATCGTCAACAACA		
*cel74a*_SC_F	ACGAGTTGGATGTCGCGCTTTG	1611 (bp)	5′ reg
*Pyr4*_5′rev	ACAAGGACTGAGGATGTTCGG		
*cel74a*_SC_R	CAGCCGACAACACCACAACTC	1830 (bp)	3′ reg
*Pyr4*_3′fwd	TGCAACAACACGCGATGG		
*cel74a*_F_ORF	CACGAACAGATATTGGCGGG	1078 (bp)	*cel74a* screenir
*cel74a*_R_ORF	GTTGGGTCAATCTCGAGAGACTC		
*cel74a*_ RT_ F	CGGTTGTATCGTACCTATTCCG	---	q
cel74a _RT_R	TGATGTCTTTCCAAGTTCCCC		
*Sar1*_F	TGAGAGCGGTGGTATCCACG	---	q
*Sar1*_R	GGTACCACCAGACATGACAATGTT		
*Bxl1*_F	CAAGTCTGGAATGAGGCTCTG	---	q
*Bxl1*_R	TGATGTCGGCAATCTGGTG		
*Cel7a*_F	CCGAGCTTGGTAGTTACTCTG	---	q
*Cel7a*_R	GGTAGCCTTCTTGACTGAGT		
*Cel7b*_F	CCCTCAACACTAGCCACCAG	---	q
*Cel7b*_R	AGGTCTTGGAGGTGTCAACG		
*Cel3a*_F	CTGTACATCACCTACCCATC	---	q
*Cel3a*_R	TAGCTGAGATCTCGTCGTC		
*Xyn1*_F	GGCCAAATTATCGTCAACTGTC	---	q
*Xyn1*_R	TCTGTCTTTTGGGCTTGGAG		
*Xyn2*_F	TGTCAACGAGCCTTCCATC	---	q
*Xyn2*_R	TCTGCACAGTAACAGTCCCG		
*Xyn3*_F	AAGTCATCCGCACCCATG	---	q
*Xyn3*_R	GTTCAAAACTCACCCAAGCAC		
